# Establishment of Nephrin Reporter Mice and Use for Chemical Screening

**DOI:** 10.1371/journal.pone.0157497

**Published:** 2016-06-30

**Authors:** Junichi Tsuchida, Taiji Matsusaka, Masato Ohtsuka, Hiromi Miura, Yukiko Okuno, Katsuhiko Asanuma, Takahiko Nakagawa, Motoko Yanagita, Kiyoshi Mori

**Affiliations:** 1 TMK Project, Medical Innovation Center, Kyoto University Graduate School of Medicine, Kyoto, Japan; 2 Research Unit/Nephrological & Endocrinological Science, Sohyaku, Innovative Research Division, Mitsubishi Tanabe Pharma Corporation, Toda, Saitama, Japan; 3 Institute of Medical Science, Tokai University, Isehara, Kanagawa, Japan; 4 Department of Molecular Life Science, Division of Basic Medical Science and Molecular Medicine, Tokai University School of Medicine, Isehara, Kanagawa, Japan; 5 Medical Research Support Center, Graduate School of Medicine, Kyoto University, Kyoto, Japan; 6 Department of Nephrology, Kyoto University Graduate School of Medicine, Kyoto, Japan; 7 School of Pharmaceutical Sciences, University of Shizuoka, Shizuoka, Japan; 8 Department of Nephrology and Kidney Research, Shizuoka General Hospital, Shizuoka, Japan; Institut National de la Santé et de la Recherche Médicale, FRANCE

## Abstract

Nephrin is a critical component of glomerular filtration barrier, which is important to maintain glomerular structure and avoid proteinuria. Downregulation of nephrin expression is commonly observed at early stage of glomerular disorders, suggesting that methods to increase nephrin expression in podocytes may have therapeutic utility. Here, we generated a knockin mouse line carrying single copy of 5.5 kb *nephrin* promoter controlling expression of enhanced green fluorescent protein (EGFP) at *Rosa26* genomic locus (Nephrin-EGFP mouse). In these mice, EGFP was specifically expressed in podocytes. Next, we isolated and cultivated glomeruli from these mice, and developed a protocol to automatically quantitate EGFP expression in cultured glomeruli. EGFP signal was markedly reduced after 5 days of culture but reduction was inhibited by vitamin D treatment. We confirmed that vitamin D increased mRNA and protein expression of endogenous nephrin in cultivated glomeruli. Thus, we generated a mouse line converting *nephrin* promoter activity into fluorescence, which can be used to screen compounds having activity to enhance *nephrin* gene expression.

## Introduction

Slit membrane synthesized by podocytes plays an essential role to form glomerular filtration barrier in the kidney [1]. Loss in the function and numbers of podocytes is a key event in various renal disorders, leading to proteinuria, glomerulosclerosis and, eventually, end-stage renal disease [2]. To alleviate podocyte injury is one of major strategies to treat chronic kidney disease, but still efficient treatment options are limited [3]. Nephrin is a critical component of glomerular filtration barrier [4]. Downregulation of nephrin expression is commonly observed at early stage of glomerular disorders [3, 5], suggesting that methods to increase nephrin expression in podocytes may have therapeutic utility.

Vitamin D has been known to increase nephrin expression in cultured mouse podocytes [6]. In renal precursor cells derived from human kidney cortex [7] or human amnion [8], vitamin D upregulates nephrin expression and induces differentiation into podocytes. Furthermore, recent reports have shown that vitamin D ameliorates proteinuria and glomerular lesions in murine models of diabetic nephropathy [9, 10] or puromycin-induced nephrosis [11]. These findings suggest that screening of compounds having activity to enhance nephrin expression may lead to identification of a new therapeutic reagent to combat glomerular disorders.

Screening chemicals using gene promoter activity as an index is often performed using cultured cells transfected with a reporter plasmid encoding a fluorescent protein or an enzyme at the downstream of promoter sequence. However, the copy numbers or genomic locations of inserted gene cannot be controlled by gene transfer methods such as lipofection or electroporation. Therefore, it is important to analyze several independent clones to verify reproducibility. Transgenic (Tg) animals are quite useful to analyze a role of gene in vivo, but they also have the same problems of Tg copy numbers and integration sites and also a concern about unexpected disruption of unrelated gene. Indeed, introducing too much reporter DNA into cells may consume up transcription factors, resulting in unphysiological responses. Therefore, to strictly study developmental, spatio-temporal or pathophysiologic regulation of gene expression in vivo, gene knockin method by homologous recombination has been used to insert reporter gene under control of endogenous promoter [12], but these experiments are time consuming.

We invented a method to insert a single copy of DNA cassette, uni-directionally, into a specific genomic locus by direct injection of DNA construct into fertilized eggs utilizing the Cre-loxP system-mediated gene arrangement. This method, termed pronuclear injection-based targeted transgenesis (PITT), bypasses a use and screen of embryonic stem cells [13, 14], and can markedly reduce potential interference from enhancer or silencer sequences surrounding the inserted gene.

In this study, we report establishment of a mouse cell line expressing enhanced green fluorescence protein (EGFP) under *nephrin* promoter using PITT method, and its use to screen compounds with activity to enhance *nephrin* expression.

## Materials and Methods

### Chemicals and media

Chemical reagents and media were purchased from Nacalai Tesque (Kyoto, Japan) unless otherwise described.

### Generation of nephrin reporter mice using PITT method

**Fig 1:**
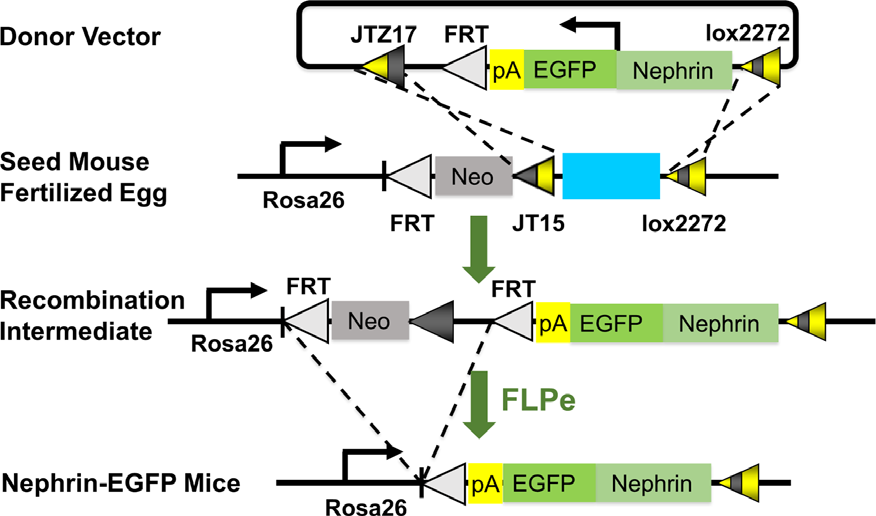
Schematic diagram of pronuclear injection-based targeted transgenesis (PITT). The donor vector is co-injected with Cre expression plasmid into the pronuclei of fertilized eggs obtained from seed mice. As a result of site-specific recombination between mutant loxPs (lox2272, JTZ17 and JT15) by Cre enzyme, one copy of the donor vector is integrated into *Rosa26* genomic locus to yield recombination intermediate mice. Extra sequence containing selection marker (Neo) and vector sequence is removed by flippase (FLP)–FLP recombinase target (FRT) gene rearrangement, resulting in generation of Nephrin-EGFP mice. Nephrin, *nephrin* promoter sequence; EGFP, EGFP cDNA; pA, poly A signal; Neo, neomycin resistance gene cassette; FLPe, FLPe-overexpressing deleter mouse.

Donor vector pBEJ, carrying nephrin promoter sequence, EGFP cDNA, polyA signal and FRT, was generated as described previously [13–15]. Its insert was flanked with 2 loxP mutant sequences, lox2272 and JTZ17, which allow directional gene recombination by Cre recombinase (Fig 1). Cre expression plasmid (5 ng/μl) and pBEJ (10 ng/μl) were co-injected into pronuclei of fertilized eggs obtained from a seed mouse strain, *Gt(ROSA)26Sor*^*tm1Maoh*^ (also termed as TOKMO-1) [13]. Seed mice carry mutant loxP sequences (lox2272 and JT15), neomycin resistance gene cassette and flippase (FLP) recombinase target (FRT) sequence at intron 1 of *Rosa26* locus. FLPe Tg mice (RBRC01835, RIKEN BioResource Center, Ibaragi, Tsukuba, Japan) were used to delete extra sequence containing neomycin cassette and vector sequence flanked with FRT [16]. Functional analysis of the reporter mouse line was carried out after backcrossing to C57BL/6N mice (Charles River Laboratories Japan, Yokohama, Japan) for 3 generations.

Animal care and procedures were approved by Institutional Animal Care and Use Committees of Tokai University (permit numbers 121007, 132013) and Kyoto University Graduate School of Medicine (Med Kyo 13116). Mice were maintained on a 12 hour light/dark cycle with free access to standard diet (F-2, Oriental BioService, Kyoto, Japan) and water. All mice were examined twice a week and were physically healthy. Overall mortality rate of mice was 2%. Cervical dislocation was used for euthanasia.

For genotyping of EGFP-Nephrin mice, genomic DNA extracted from tail was amplified with Thunderbird Probe qPCR Mix (Toyobo, Osaka, Japan) and analyzed by StepOnePlus Real-Time PCR System (Thermo Fisher Scientific, Waltham, MA, USA). Quantitation was carried out by ΔΔCT method. Amount of *EGFP* DNA was normalized by that of *Mafb* DNA (which has no introns) as internal control. PCR primers and probes used are shown in Table 1.

**Table 1:** Primer and probe sequences for qPCR of genomic DNA and cDNA.

*Gene*		Nucleotide Sequence
	P	CCACCTACGGCAAGCTGACCCTGAAGT
*EGFP*	F	GGCCACAAGTTCAGCGTGTC
	R	GTAGGTCAGGGTGGTCACGA
	P	CCTTGCCCTGGCCCAGACTCCCTATT
*Mafb*	F	TCTCCTGAGTTCTTTCTGTGAGTC
	R	CTTCCTCCCTCTAGCTCAAGTCA
	P	AGGGCTCACGCTCACAACCTTCAGC
*Nphs1*	F	AGACCACACCAACATCCAGC
	R	AAGCCAGGTTTCCACTCCAG
	P	TTTGATGCCCCAAATACAGGTCACTGCA
*Nphs2*	F	GGAAGAGCATTGCCCAAGATG
	R	AGCCTCACATCCTTAATTTCAGTTC
	P	AACTAAGAACGGCCATGCACCACCACC
*18S*	F	GACAGATTGATAGCTCTTTCTCGA
	R	CGGAATTAACCAGACAAATCGCTC

Probes (P) were modified by FAM and TAMRA at 5’ and 3’ ends, respectively. F, forward primer. R, reverse primer.

### Reverse transcription-PCR for gene expression analysis

Total RNA was extracted from samples using RNeasy Plus Mini Kit (Qiagen, Hilden, Germany) and cDNA was synthesized by ReverTra Ace qPCR RT Master Mix with gDNA Remover (Toyobo, Osaka, Japan). Gene expression levels of *nephrin* (*Nphs1*) and *podocin* (*Nphs2*) were examined by quantitative PCR (qPCR) with Thunderbird Probe qPCR Mix and StepOnePlus Real-Time PCR System, and normalized by 18S ribosomal RNA levels (Table 1) [17, 18].

### Immunostaining of tissues

Kidney tissue in 1mm thickness was fixed with 4% paraformaldehyde (PFA) at 4°C for 30 min and incubated at 4°C overnight with 20% sucrose in phosphate-buffered saline (PBS). Next day, tissues were frozen in Tissue-Tek O.C.T. compound (Sakura Finetek Japan, Tokyo, Japan) and stored at -80°C until analysis. Frozen tissues were sliced with cryostat (CM1950, Leica Biosystems, Wetzlar, Germany) at 4 μm thickness. For immunofluorescence [17], kidney sections or cultured tissues were incubated with PBS containing 10% normal donkey serum (Dako, Agilent Technologies, Santa Clara, CA, USA) and 0.3% Triton X-100 for 30 min, washed and incubated with primary antibodies (1:200 dilution) at 4°C overnight: goat anti-mouse nephrin (R&D Systems, Minneapolis, MN, USA), rabbit anti-mouse/human podocin (against carboxyl terminal 17 amino acid polypeptide) [19], rat monoclonal anti-mouse platelet-derived growth factor receptor β (PDGFRβ; cloneAPB5, BioLegend, San Diego, CA, Japan) and rat monoclonal anti-mouse platelet endothelial cell adhesion molecule-1 (PECAM-1 or CD31; clone MEC 13.3, BD Biosciences, Franklin Lakes, NJ, USA). Primary antibodies were detected with Alexa Fluor 568-conjugated secondary antibodies (1:200, room temperature for 60 min; Thermo Fisher Scientific) and mounted with Vectashield (Vector Laboratories, Burlingame, CA, USA). Plane and fluorescence pictures were taken by All-in-One microscopy (BZ-X710, Keyence, Itasca, IL, USA).

For immunohistochemistry of EGFP, kidney tissues were fixed in 4% PFA at 4°C overnight and embedded in paraffin. Renal sections of 2 μm were deparaffined, incubated in 0.3% hydrogen peroxide/methanol for 15 minutes, hydrated, and incubated with 0.1% trypsin at 37°C for 10 minutes. After rinsing in PBS, sections were incubated with 10% normal goat serum, followed by rabbit polyclonal anti-GFP antibody, which recognizes EGFP (1:500, MBL 598, MBL). Primary antibody was visualized with biotin-conjugated goat anti-rabbit IgG (1:200, Jackson ImmunoResearch), Vectastain Elite ABC kit (Vector) and 3,3-diaminobenzidine tetrahydrochloride (Dako). Nuclei were counterstained with methyl blue.

### Isolation and primary culture of glomeruli

Glomeruli were isolated from 8–20 week-old C57BL/6J or Nephrin-EGFP mice using magnetic beads as described [20]. Under sodium pentobarbital anesthesia (Somnopentyl, Kyoritsu Seiyaku, Tokyo, Japan), Dynabeads M-450 Tosylactivated (8 x 10^7^, Thermo Fisher Scientific) dissolved in 30 ml of Hanks’ balanced salt solution (HBSS) were injected through the left ventricle. The kidneys were removed, minced, and digested in HBSS containing collagenase A (1 mg/ml, Roche Diagnostics, Penzberg, Germany) and deoxyribonuclease I (100 U/ml, Roche Diagnostics) at 37°C for 30 minutes with agitation at 120 rpm. Digested tissue was passed through 100 μm cell strainer (Falcon, Corning, NY, USA), and glomeruli were collected by magnetic stand (Thermo Fisher Scientific) and washed three times with PBS containing 1% globulin-free bovine serum albumin.

Isolated glomeruli were seeded on 96-well optical plates (Thermo Fisher Scientific) coated with rat type I collagen (0.3 mg/ml, Corning) at a density of 100–150 glomeruli per well (day 0) in Dulbecco's Modified Eagle's Medium (with 4.5 g/l glucose, DMEM) containing 10% fetal bovine serum (FBS, Gibco, Thermo Fisher Scientific) unless otherwise described, and incubated at 37°C with 5% CO_2_ without passage. After 3 days (on day 3), medium was changed to DMEM containing 0.5% FBS, and 1α,25-(OH)_2_ vitamin D_3_ (Cayman Chemical, Ann Arbor, MI, USA), all trans-retinoic acid (Sigma, St. Louis, MO, USA), screening compound or vehicle was added. Cultivation was continued for 2 more days.

### Chemical library screening

Pharmacologically active compound libraries were obtained from Prestwick Chemical (Illkirch-Graffenstaden, France), Calbiochem (Merck Millipore, Darmstadt, Germany) and Selleck Chemicals (Houston, TX, USA) through Medical Research Support Center, Graduate School of Medicine, Kyoto University. Stock solutions (10 mM) were prepared in 100% dimethly sulfoxide (DMSO) and were arrayed in 96 well plates. Chemical stocks were first diluted 1:10 with DMSO and further 1:20 with DMEM containing 0.5% FBS. Diluted chemicals (40 μl) were added into wells (160 μl) containing glomerular tissues to give final concentrations of 10 μM chemical, 1% DMSO and 0.5% FBS.

### Quantitative assay of fluorescence

After cultivation, primary culture of glomerular tissue was fixed with 4% PFA at 4°C for 30 min and stained by immunofluorescence, if appropriate, and also stained with 4',6-diamidino-2-phenylindole (DAPI, 500 ng/ml, Dojindo, Kumamoto, Japan). Fluorescence intensities of tissues immersed in 100 μl PBS were measured by ArrayScan VTI HCS Reader (Thermo Fisher Scientific) as described previously [21] using 5x objective (numerical aperture 0.25) through 3 channels: channel 1 (BGRFR 386–23 for DAPI), channel 2 (485–20 for EGFP) and channel 3 (549–15 for Alexa Fluor 568). Images were analyzed by HCS Studio 2.0 Cell Analysis Software with Cell Health Profiling Algorithm (Thermo Fisher Scientific). Glomeruli were identified as clusters of DAPI signals whose sizes were larger than a threshold value described in the Result section. Regions of interest (ROI) were set to glomeruli and signals of respective glomeruli at channel 2 or 3 were recorded. Data from 4 fields covering majority of a single well were combined and the mean value among glomeruli of each well was calculated.

### Statistical analysis

Results are expressed as mean±SEM. Data were analyzed by 2-tailed Student’s t test or ANOVA. Statistical significance was defined as P<0.05.

## Results

### Generation of nephrin reporter mice using PITT method

**Fig 2:**
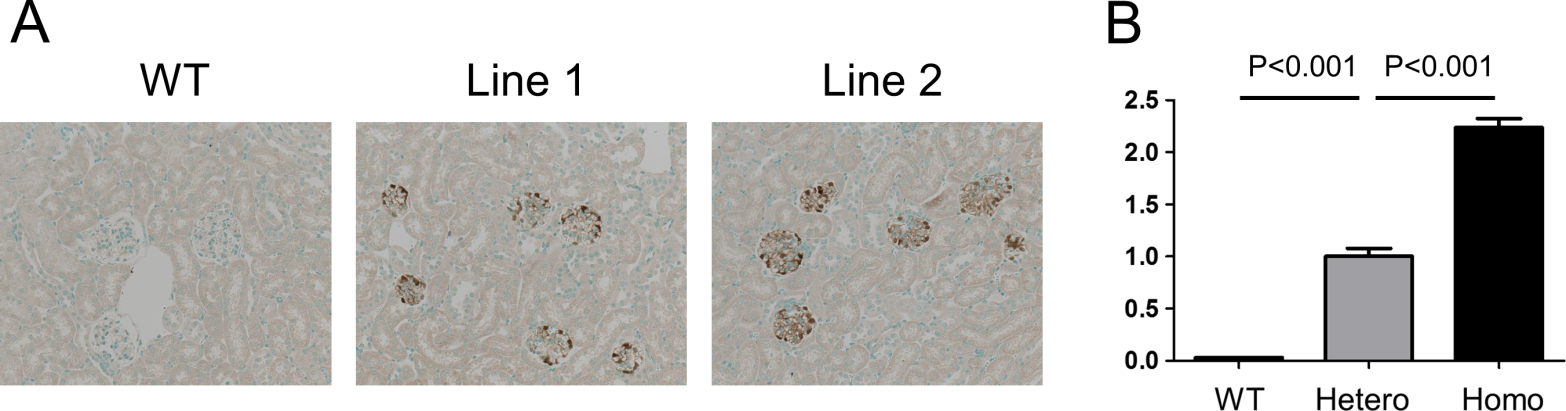
EGFP expression and genotyping of Nephrin-EGFP mice. (**A**) EGFP protein was expressed preferentially at periphery of glomeruli in two lines of Nephrin-EGFP mice by immunohistochemistry using anti-GFP antibody. (**B**) Genotyping of wild-type (WT), heterozygous (Hetero) and homozygous (Homo) mice by genomic qPCR. Amount of EGFP genomic DNA detected in WT mice was less than 1% of heterozygous mice. Mean±SEM of n = 4–8. Comparison was carried out by unpaired t test. Magnification, 10x.

A 5.5 kb *nephrin* promoter sequence has been shown to target gene expression specifically into podocytes of Tg mice [15], and nephrin-Cre mice have been successfully used to make podocyte-specific conditional knockout animals [22, 23]. A donor vector carrying Nephrin+EGFP expression cassette and a Cre expression vector were co-injected into fertilized eggs isolated from the seed mice to obtain knockin mice carrying a single copy of Nephrin+EGFP cassette at the *Rosa26* locus in the opposite direction to *Rosa26* transcription[13, 14] (Fig 1). Of 53 pups obtained, two mice showed expected gene recombination at the *Rosa26* locus in their tail DNA. These two lines were mated with FLPe mice to remove extra sequence [16] and EGFP expression was confirmed in both lines preferentially at the periphery of glomeruli by immunohistochemistry (Fig 2A). Line 1 was named Nephrin-EGFP mice, and further analyzed. By genomic qPCR, wild-type, heterozygous and homozygous Nephrin-EGFP mice could be distinguished (Fig 2B). Heterozygotes were used to study regulation of EGFP expression.

### Renal localization of EGFP expression

**Fig 3:**
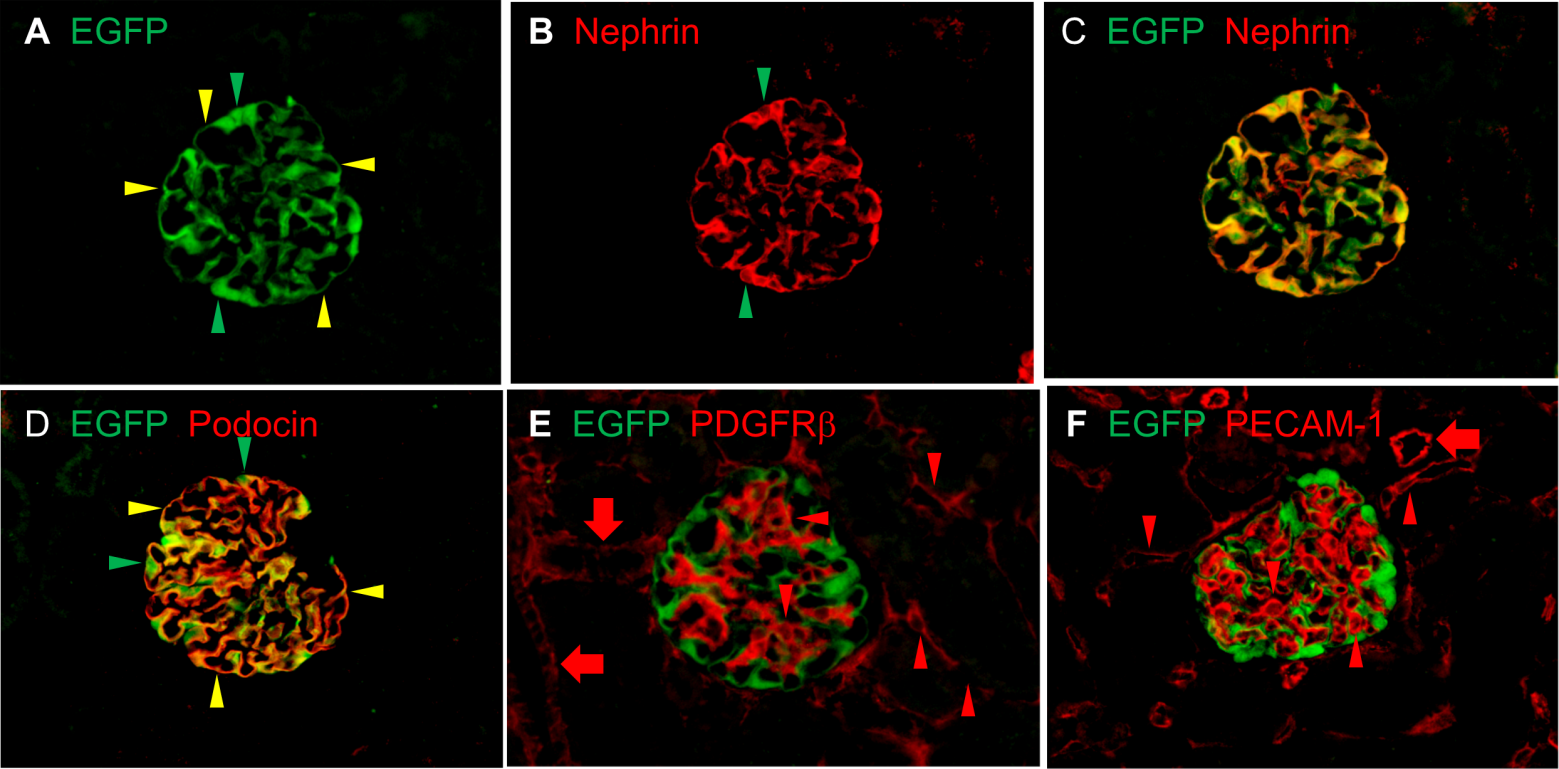
Protein expression of EGFP and glomerular cell markers in Nephrin-EGFP mice. (**A-C**) EGFP and nephrin were co-expressed along glomerular capillaries (yellow arrowheads). Cell bodies of podocytes located at the outer surface of glomerular capillaries (green arrowheads) also expressed EGFP (**A**) but nephrin expression was spared (**B**). (**D**) Similarly, EGFP and podocin were expressed along glomerular capillaries (yellow arrowheads). Podocyte cell bodies expressed EGFP but not podocin (green arrowheads). (**E**) PDGFRβ was expressed at glomerular and peritubular capillaries (arrowheads) and walls of arterioles (arrows), and did not co-localize with EGFP. (**F**) PECAM-1 was expressed along glomerular and peritubular capillaries (arrowheads) and inner surface of arterioles (arrow), and did not merge with EGFP. Magnification, 40x.

By immunofluorescence, EGFP expression co-localized well with endogenous nephrin and podocin, which are podocyte-specific molecules (Fig 3). On the other hand, cells stained by antibodies against PDGFR β (mesangial cell marker) or PECAM-1 (endothelial cell marker) did not express EGFP. Outside of glomeruli, no EGFP expression was observed. These findings verified that EGFP was specifically expressed in podocytes of Nephrin-EGFP mice.

### Cultivation of glomeruli from Nephrin-EGFP mice

**Fig 4:**
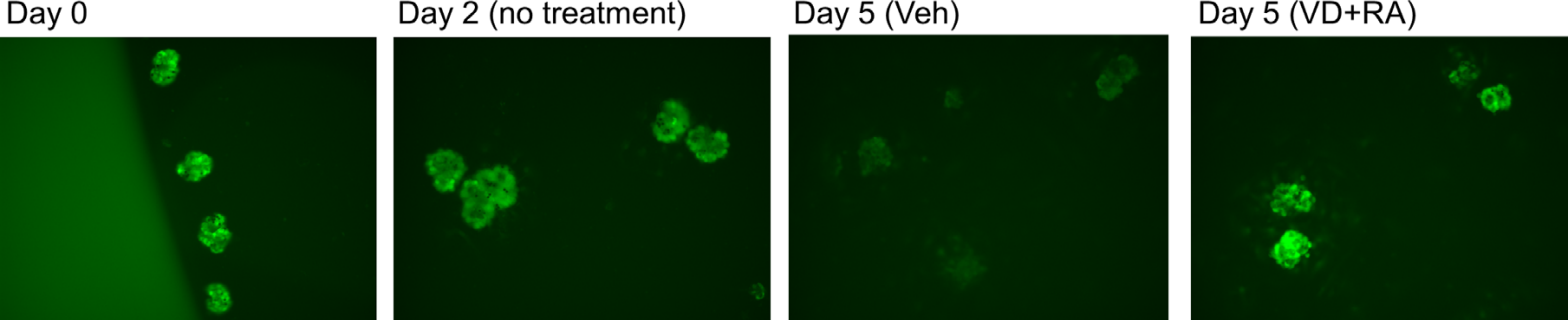
EGFP expression in cultured glomeruli from Nephrin-EGFP mice. On Day 2, EGFP expression remained mainly along the periphery of glomeruli, but was markedly reduced on day 5. FBS concentration was reduced from 10% to 0.5% on day 3. Treatment with 1α,25-(OH)_2_ vitamin D_3_ (VD, 50 nM) and all trans-retinoic acid (RA, 1 μM) for the last 2 days induced EGFP expression. Veh, vehicle. Magnification, 10x.

**Fig 5:**
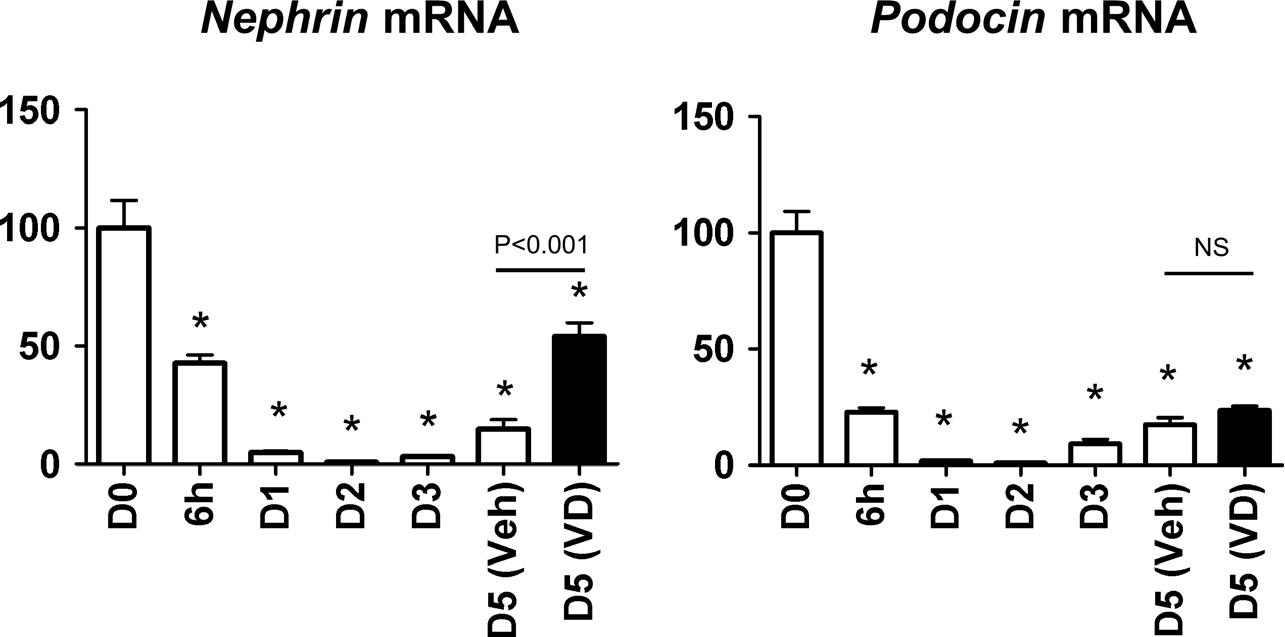
Expression of *nephrin* and *podocin* mRNA in cultured glomeruli. Gene expression was studied on days 0–5 (D0-D5) and at 6 hours (6h). Vitamin D (VD, 50 nM) or vehicle (Veh) was added for the last two days. Expression levels were normalized by 18S ribosomal RNA and levels at D0 were defined as 100%. Mean±SEM of n = 5. Treatment with VD significantly upregulated *nephrin* expression by unpaired t test. NS, not significant. *P<0.001 vs D0 by one-way ANOVA with Dunnett’s post hoc test.

By magnetic bead method, approximately 10,000–20,000 glomeruli were obtained per mouse (95% purity by light microscopy) as previously described [20]. When glomeruli from Nephrin-EGFP mice were cultivated, EGFP fluorescence persisted at least for 2 days on the surface of glomeruli, but was markedly reduced within 5 days (Fig 4). On the other hand, after 1 or 2 days, *nephrin* mRNA level was decreased to 4.9% of the initial level on day1, further decreased to 0.8% on day 2, and partially recovered to 14.9% on day 5 (Fig 5). Apparent difference in the time course between EGFP fluorescence and *nephrin* mRNA was likely caused by much longer half life of EGFP molecule compared to that of endogenous *nephrin* mRNA. *Podocin* mRNA of cultured glomeruli showed similar changes: 1.8%, 0.9% and 17.6% on days 1, 2 and 5, respectively (Fig 5).

Previous reports showed that nephrin expression in cultured podocytes or renal precursor cells was increased by treatment with 10–100 nM vitamin D and 1 μM retinoic acid [6–8]. In glomeruli from Nephrin-EGFP mice, addition of vitamin D+retinoic acid increased EGFP fluorescence (Fig 4), thus validating our reporter system.

### Automated quantitation of EGFP signals

**Fig 6:**
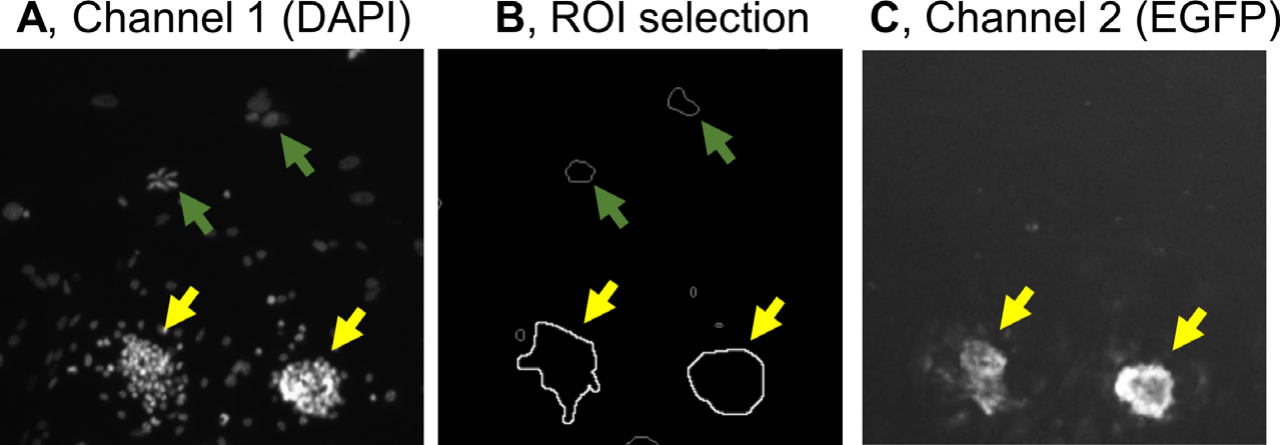
Quantitative evaluation of EGFP signals in glomerular areas. (**A**) Glomeruli appeared as clusters of DAPI signals. (**B**) DAPI clusters having areas larger than > 2000 μm^2^ were recognized as glomeruli and ROI were set to such glomerular areas (yellow arrows), whereas smaller clusters were excluded from ROI (green arrows). (**C**) EGFP signals were measured only in ROI. Magnification, 10x.

To detect glomeruli, cultivated material was fixed and stained with a nuclear marker DAPI. Blue signals by DAPI were scattered in podocyte outgrowth, but were clustered in glomeruli (Fig 6A). The sizes of DAPI clusters were automatically calculated. When a threshold of > 2000 μm^2^ was selected, recognition of glomeruli from migrated podocytes was best performed and matched well with visual judgement (Fig 6B). This threshold seems reasonable since it is consistent to a diameter of > 45 μm used in classical sieving method for mouse glomerulus isolation [24]. After setting ROI to glomerular areas, EGFP signals in glomeruli were quantitated as green fluorescence (Fig 6C).

**Fig 7:**
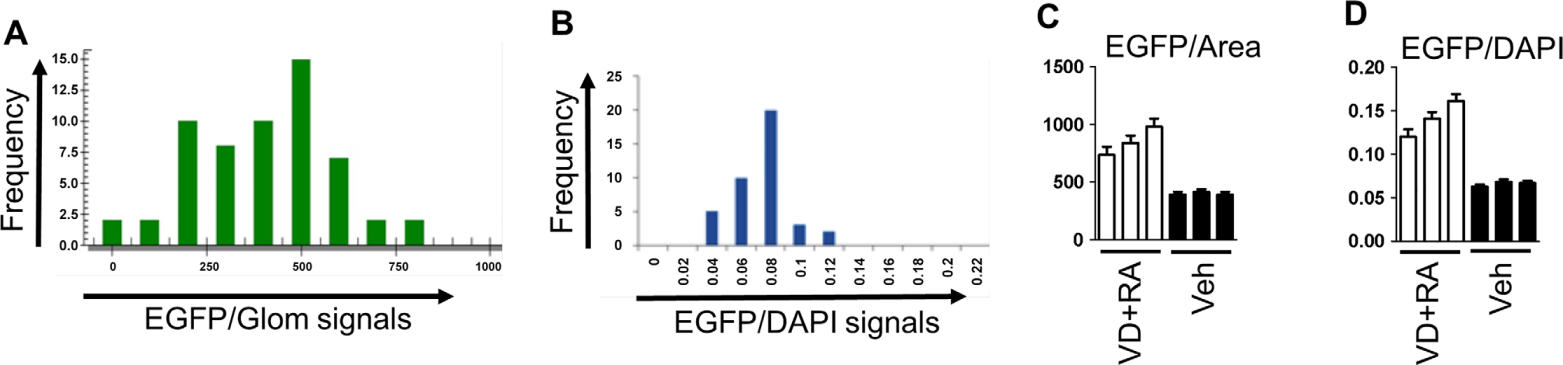
Histogram of EGFP signals of respective glomeruli. (**A**) In a single well, total EGFP signal of each glomerulus was calculated individually, and fractionated as histogram. (**B**) EGFP signal per DAPI signal was calculated and analyzed. (**C**) Each bar indicates mean±SEM of EGFP/Area among several dozens of glomeruli from a single well (Fig 8A). Vitamin D+retinoic acid (VD+RA) or vehicle (Veh)-treated glomeruli were studied in triplicate wells. (**D**) Each bar indicates mean±SEM of EGFP/DAPI of a well.

After 5 days of culture, some glomeruli showed preserved structure, while other glomeruli appeared destructed or melted by escape of cells from glomeruli (Fig 6A). As a consequence, total EGFP signals (Fig 7A) and the numbers of DAPI-positive cells (Fig 6A) of individual glomeruli were highly variable even in a single well. Consistently, when EGFP signal per DAPI signal was calculated for each glomerulus, distribution of histogram was much concentrated (Fig 7B). When normalization of EGFP signal by area and that by DAPI signal were compared, they gave very similar patterns (Fig 7C and 7D). Therefore, simple normalization by area was chosen, and the average of EGFP signal/glomerular area among whole glomeruli from each well was used as a representative value for that well.

**Fig 8:**
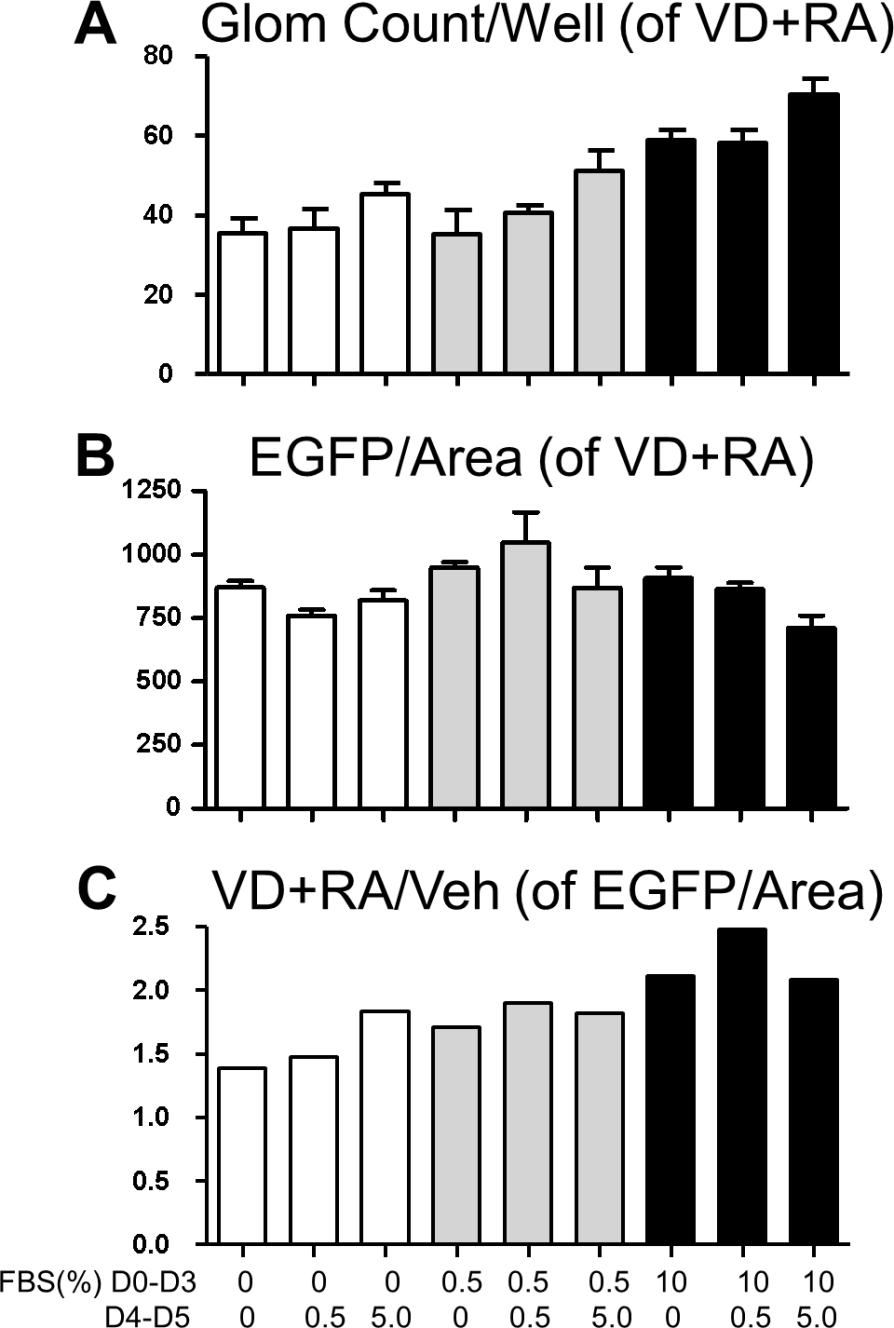
Effects of FBS upon EGFP signals. Glomeruli from Nephrin-EGFP mice were cultivated for the first 3 days (D0-D3) and the last 2 days (D4-D5) with DMEM containing different concentrations of FBS and fluorescence was examined on D5. Vitamin D (50 nM, VD)+retinoic acid (1 μM, RA) or vehicle (Veh) was added during D4-D5. (**A**) Glomerular count per well of glomeruli given VD+RA during D4-D5. Mean±SEM of n = 3. (**B**) EGFP signal per glomerular area. N = 3. (**C**) Effects of VD+RA upon EGFP/Area signals. The average value from wells treated with VD+RA was divided by the average value from Veh-treated wells.

Next, FBS concentration for chemical screening was optimized (Fig 8). After isolation from Nephrin-EGFP mice, glomeruli were seeded at a density of 100–150 glomeruli per well and cultivated for 3 days in DMEM containing 0%, 0.5% or 10% FBS (Days 0–3). By changing medium to fresh DMEM with 0%, 0.5% or 5% FBS on day 3, approximately half of glomeruli were removed. On day 5, cultured materials were fixed and stained with DAPI. At this point, approximately one third of glomeruli of the initial number were retained on plates (with some variations) and EGFP fluorescence was quantitated. Culturing glomeruli with 10% FBS for the first 3 days (days 0–3) increased the recovery of glomeruli by 30% compared to 0% or 0.5% FBS (Fig 8A). Cultivation with 5% FBS during the last 2 days (days 4–5, in the presence of vitamin D plus retinoic acid) further increased recovery by 20% compared to 0% or 0.5% FBS during days 4–5. When EGFP signals were measured as EGFP/Area, cultivation with 5% FBS during days 4–5 reduced the signals by 20% compared to 0.5% FBS during days 4–5 (after incubation with 0.5% or 10% FBS during days 0–3), presumably due to enhanced outgrowth and escape of podocytes from glomeruli by 5% FBS during days 4–5 (Fig 8B). As a consequence, 10% FBS on days 0–3 plus 0.5% FBS on days 4–5 gave the largest difference between vitamin D+retinoic acid vs vehicle treatment in EGFP/Area values (by more than 2-fold, Fig 8C), and these FBS conditions were selected.

### Performance of Nephrin-EGFP mouse glomeruli in culture system

**Fig 9:**
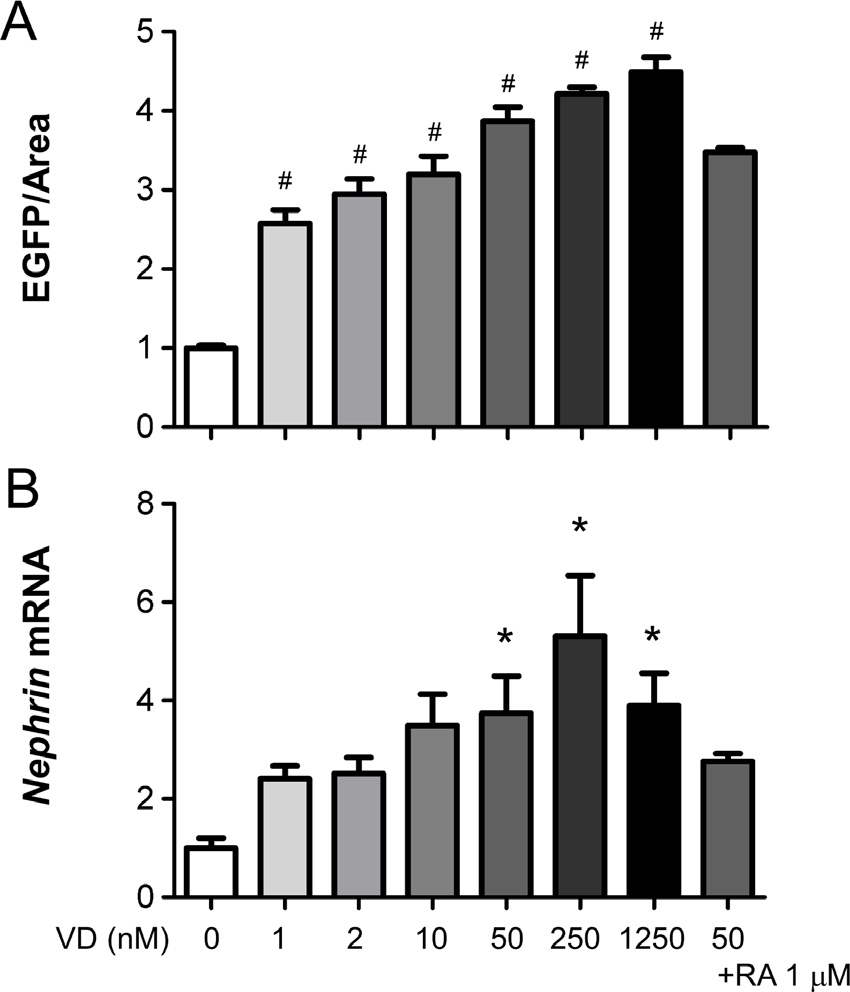
Dose-dependent induction of EGFP signals and *nephrin* mRNA expression by vitamin D. Cultured glomeruli from Nephrin-EGFP mice were treated with 1–1250 nM of vitamin D (VD), 1 μM retinoic acid (RA) or vehicle (0 nM) for the last 2 days and analyzed on day 5. (**A**) EGFP/glomerular area. Mean background signal of wild-type glomeruli was subtracted. (**B**) *Nephrin* mRNA expression. Expression levels were normalized by 18S ribosomal RNA. Each bar indicate mean±SEM of triplicate wells. The level of vehicle was defined as 1.0 unit. ^#^P<0.001 or *P<0.05 vs vehicle by one-way ANOVA with Bonferroni’s multiple comparison test.

First, the effects of various vitamin D concentrations were tested. EGFP signals were dose-dependently increased by vitamin D: 2.6-fold by 1 nM, 3.9-fold by 50 nM and 4.5-fold by 1250 nM (Fig 9). Since addition of retinoic acid (1 μM) to 50 nM vitamin D did not further increase expression of EGFP, we chose 50 nM vitamin D without retinoic acid as a positive control for glomerular EGFP assay. Treatment of glomeruli during the last two days with vitamin D in the presence of 0.5% FBS increased *nephrin* mRNA expression by 3.6-fold on day 5 compared to vehicle treatment, without significant effects upon *podocin* mRNA (Fig 5). Dose-response curves of EGFP signals and *nephrin* mRNA expression by vitamin D were considerably proportional (Fig 9).

**Fig 10:**
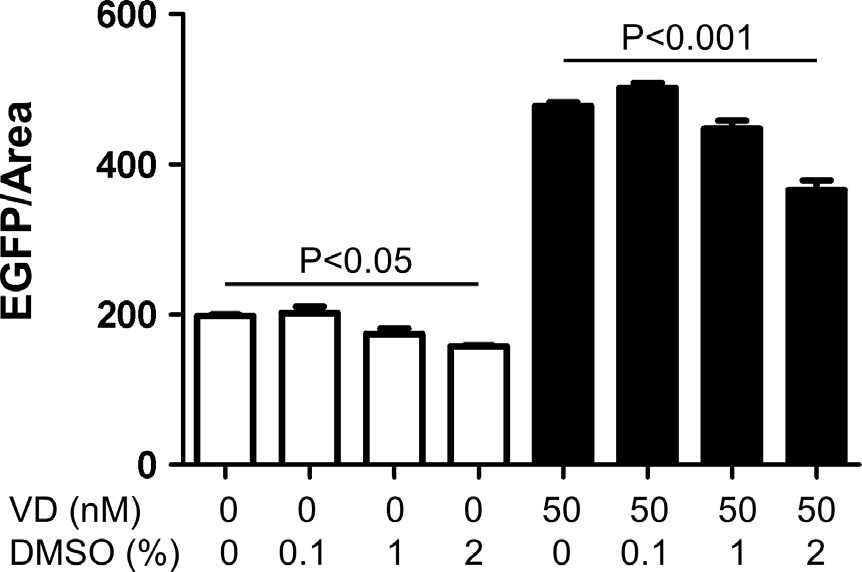
Effects of DMSO upon EGFP signals. Vitamin D (VD) or DMSO was added for the last 2 days and fluorescence was examined as EGFP/glomerular area on day 5. Mean±SEM of n = 3. Addition of 2% DMSO significantly decreased EGFP signals both in vehicle- and VD-treated glomeruli (P<0.05). Treatment with VD significantly increased EGFP signals in the presence of all concentrations of DMSO studied (0 to 2%, P<0.001). Comparison was carried out by two-way ANOVA with Bonferroni’s multiple comparison test.

We studied effects of DMSO upon chemical screening, since all reagents were first dissolved in DMSO. DMSO at 2% mildly decreased EGFP signals both in vehicle- and 50 nM vitamin D-treated glomeruli by 20% (Fig 10), but 1% DMSO showed no significant effects. Therefore, we judged that presence of 0.1–1% DMSO during screening is tolerable.

**Fig 11:**
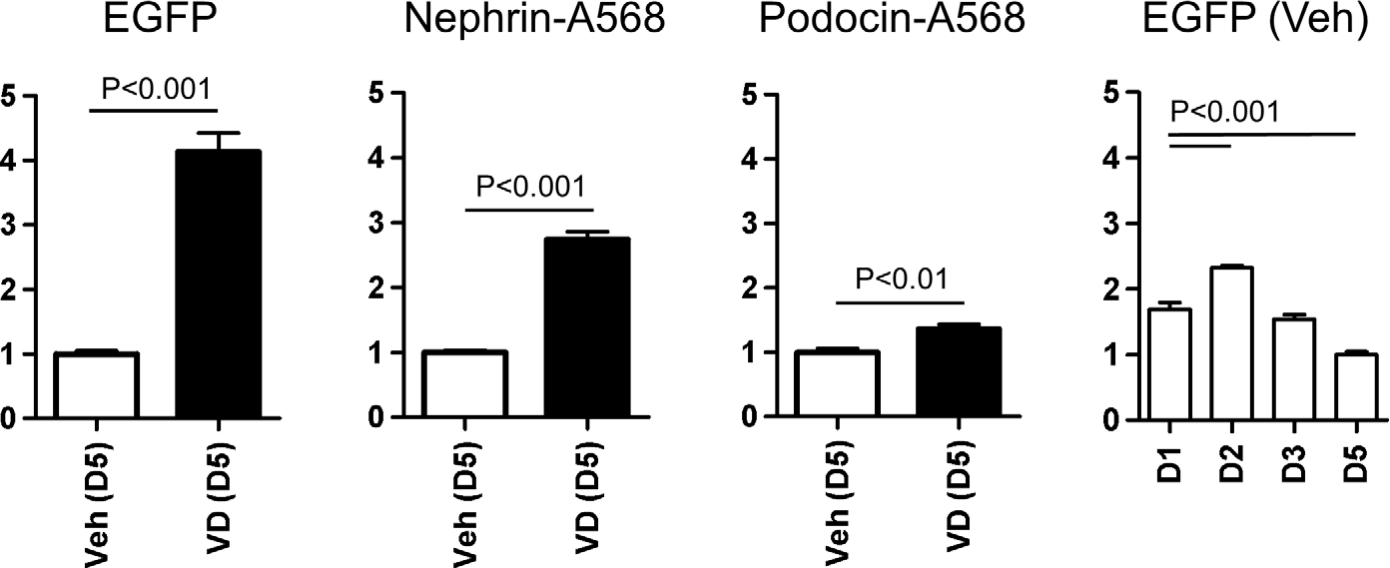
Quantitative evaluation of changes in EGFP, nephrin and podocin protein expression in glomeruli by vitamin D. Endogenous nephrin and podocin were visualized by immunofluorescence using primary antibodies and Alexa Fluor 568-conjugated secondary antibodies. Fluorescence intensity was measured on days 1–5 (D1-D5). Glomeruli were treated with 50 nM vitamin D (VD) or vehicle (Veh) for the last 2 days and examined on day 5 (D5), and the difference was studied by unpaired t test. For EGFP quantitation, mean background signal of wild-type glomeruli was subtracted and the level of Veh-treated Nephrin-EGFP glomeruli on D5 was defined as 1.0 unit. For nephrin and podocin, mean background signal of wild-type glomeruli incubated with secondary antibody alone was subtracted, respectively. Difference between EGFP signals among days was examined by one-way ANOVA with Bonferroni’s multiple comparison test. N = 5.

Endogenous nephrin protein expression in cultured glomeruli and its change by vitamin D was examined. By immunofluorescence and quantitation, nephrin protein expression was increased by 2.7-fold with vitamin D treatment, while EGFP signal was increased by 4.1-fold (Fig 11). On the other hand, podocin protein expression was only mildly increased (1.3-fold) by vitamin D, suggesting predominant effects of vitamin D upon nephrin expression. In a time course analysis, EGFP intensity was slightly elevated on day 2, and gradually decreased towards day 5.

**Fig 12:**
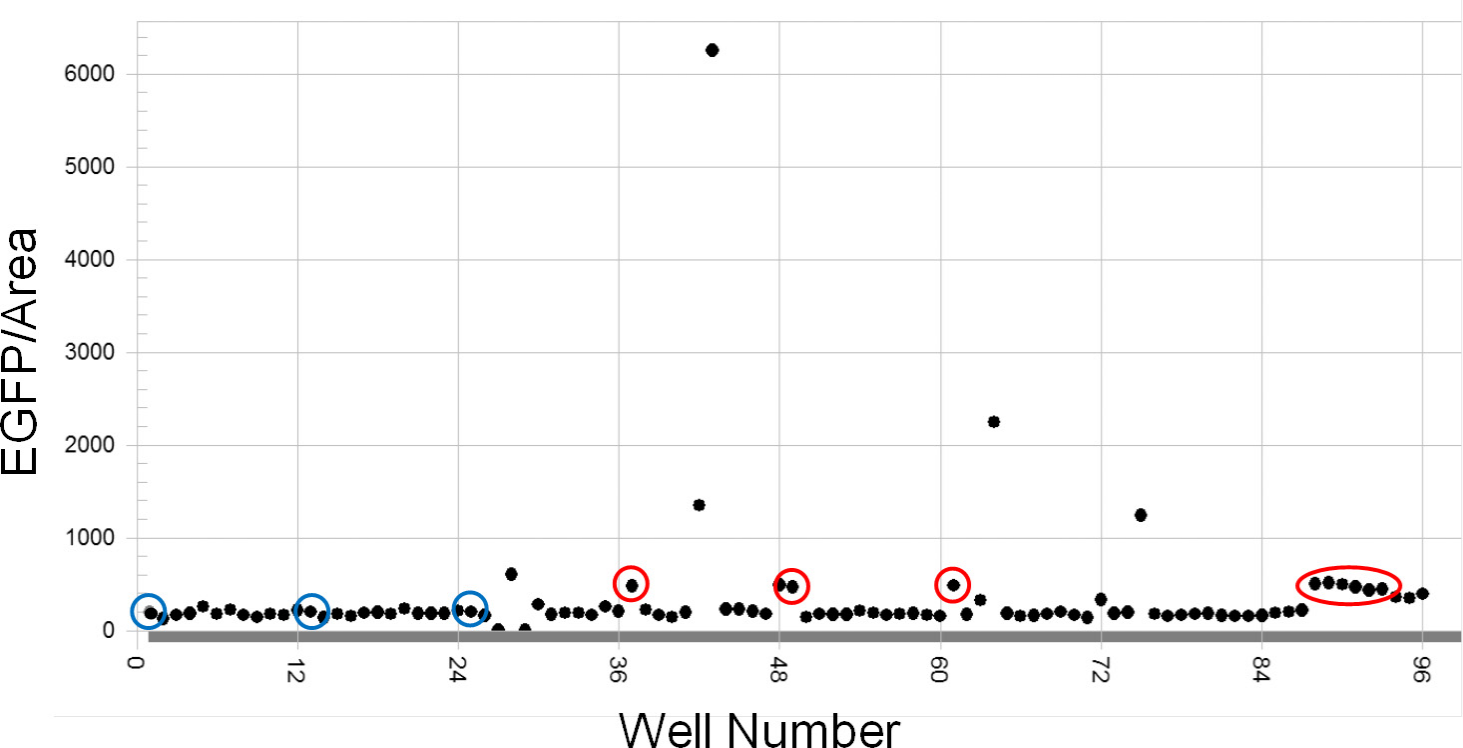
An example of chemical library screening of a plate. Y axis indicates EGFP intensity/glomerular area of each well under excitation. Wells treated with vitamin D (red circles) or vehicle (blue circles) are highlighted.

**Fig 13:**
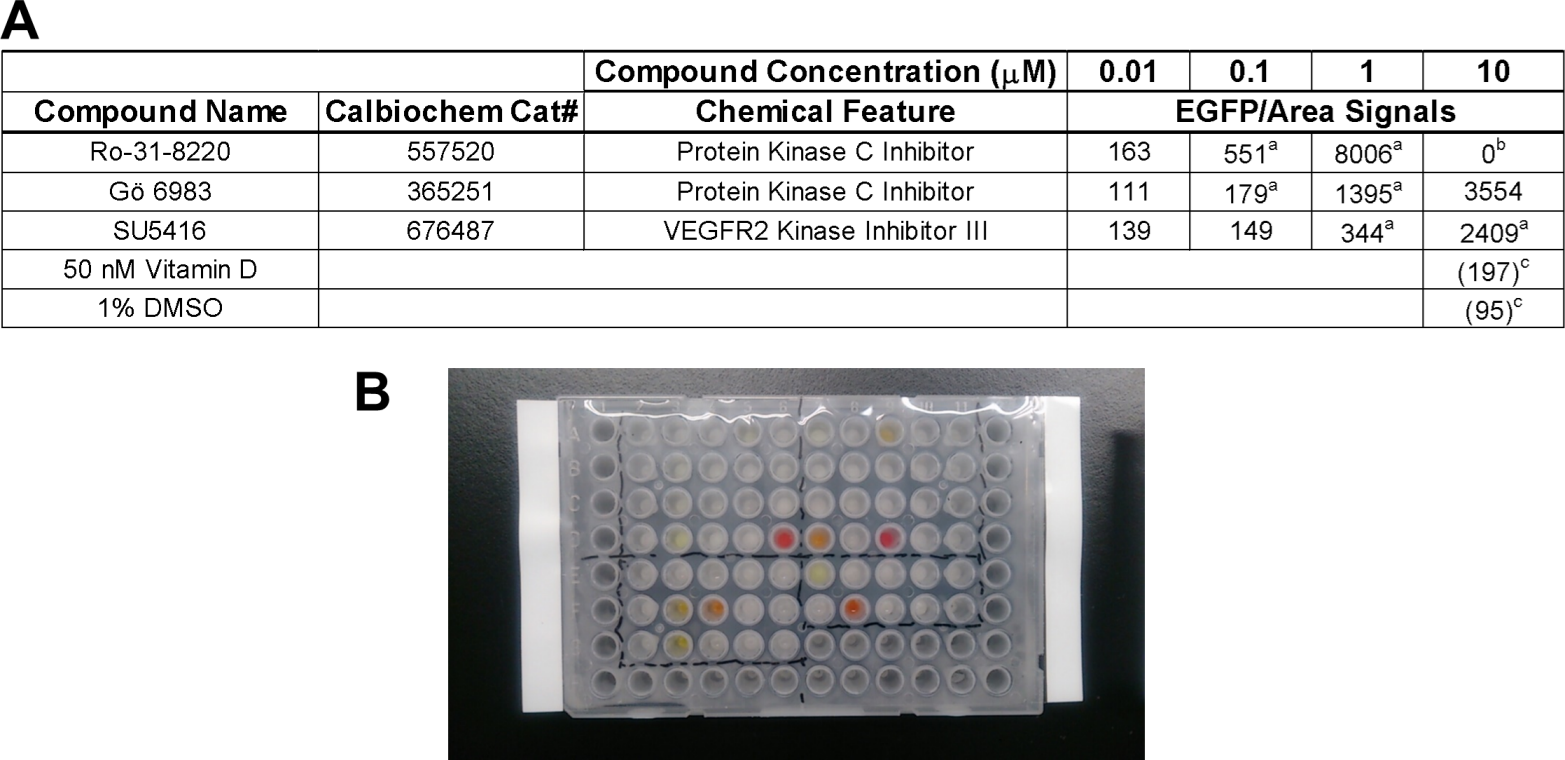
Strong and linear effects by auto-fluorescence of chemicals upon screening by EGFP signal intensity. (**A**) Cat#, catalog number. a, Pairs of compound concentrations with linear dose-response (10-times more compound giving approximately 10-times more signal), which suggest non-specific effects reflecting compound colors. b, Glomeruli treated with 10 μM of Ro-31-8220 were detached from plate likely due to drug toxicity. c, 10 nM Vitamin D and 1% DMSO served as positive and negative controls for EGFP/glomerular area signals under excitation. Background subtraction of signal by wild-type glomeruli was not carried out here. (**B**) Photograph of chemical compound stock solution (1 mM) under standard room light, showing intrinsic colors in some solutions.

An example of screening in a 96-well format is shown in Fig 12. Vitamin D-treated wells showed constantly and clearly increased EGFP fluorescence signals by excitation compared to vehicle-treated wells (Fig 12). Positive wells with similar signal levels or much stronger signals compared to vitamin D-treated wells were also found. Next, we investigated dose-response of some positive chemicals as to EGFP intensity (Fig 13). Very strong EGFP signals by several reagents were often reduced by 90% when the reagent concentrations were reduced by 90%, which was not the case with vitamin D (Fig 9). We realized that, in such cases, chemicals presumably gave very strong fluorescence in glomeruli because of the colors of reagents (which are visible even under standard room light, Fig 13), especially when they had high cell permeability to live cells. These findings suggested that studying dose-response and the color of compounds may be efficient ways to exclude false positive hits from the primary screening.

## Discussion

In the present study, we generated Nephrin-EGFP mice, which specifically express EGFP in podocytes under the control of *nephrin* promoter inserted at the *Rosa26* locus. To our knowledge, this strain is the first *nephrin* reporter line to be established by knockin strategy, instead of conventional random transgene insertion [25]. Next, we isolated glomeruli from these mice, and set up cultivation and assay which can be used to screen reagents enhancing *nephrin* transcriptional activity.

We previously showed that, unlike traditional random-insertion transgenesis approaches, the targeted transgenesis approach using PITT (insertion of a gene of interest into the *Rosa26* locus) enables to obtain Tg mice with stable, reliable, predictable, and reproducible transgene expression when constitutive CAG promoter was used [13, 14]. In the present study, we applied the PITT strategy for generation of Tg mice showing tissue-specific expression. Because both of two Nephrin-EGFP founder mouse lines identically exhibited highly podocyte-specific expression, the PITT method is useful also for production of tissue-specific Tg mice with reproducible and expected transgene expression.

Since podocytes play an essential role for maintenance of structure and function of glomeruli [1–5], cultured podocytes have been successfully used to screen chemicals possessing activities to prevent morphological change or damage of podocytes [26]. Upon cultivation of glomeruli on plates covered with type I collagen, podocytes start to migrate out as single cells [27] but substantial amount of podocytes remain upon glomeruli. For assessment of EGFP expression, we uniquely focused on podocytes attached to glomeruli, since such cells are ligated to a native scaffold, glomerular basement membrane, and located close to endogenous mesangial and endothelial cells, which might help mutual exchange of soluble factors such as vascular endothelial growth factor [25].

Our system is also unique in a point that we used freshly isolated glomeruli and podocytes without any passage. Since podocytes are terminally differentiated cells with very small proliferating capacity especially in vivo [28], primary podocytes cannot keep on growing and require immortalization procedure for propagation [29], which might alter the nature of podocytes. Further studies are required to judge whether features in our screening method provides advantage compared to methods reported so far [6, 10, 26].

There are several limitations in this work. In our culture system, certain number of podocytes are lost by day 5 (when EGFP intensity is evaluated) by podocyte outgrowth and medium change. Furthermore, we describe here that colors of the original compounds may affect the results of fluorescence-based assay. Therefore, findings in nephrin reporter assay have to be verified by evaluation of endogenous nephrin mRNA and protein expression as we did for vitamin D (Figs 5 and 11).

Vitamin D has been known to upregulate nephrin expression both in vitro and vivo, and to inhibit glomerular injury [6, 9–11]. Using Nephrin-EGFP glomeruli, we could show that vitamin D increases EGFP fluorescence intensity as a positive control essential for chemical library screening.
